# The intake of flavonoids, stilbenes, and tyrosols, mainly consumed through red wine and virgin olive oil, is associated with lower carotid and femoral subclinical atherosclerosis and coronary calcium

**DOI:** 10.1007/s00394-022-02823-0

**Published:** 2022-03-07

**Authors:** Henry Montero Salazar, Raquel de Deus Mendonça, Martín Laclaustra, Belén Moreno-Franco, Agneta Åkesson, Pilar Guallar-Castillón, Carolina Donat-Vargas

**Affiliations:** 1 Department of Preventive Medicine and Public Health and Microbiology, Universidad Autónoma de Madrid, CEI UAM+CSIC+ IdiPaz, Madrid, Spain; 2 Department of Clinical and Social Nutrition. School of Nutrition, Universidade Federal de Ouro Preto, Ouro Preto, Brasil; 3 Insituto de Investigación Sanitaria (IIS) Aragón, Hospital Universitario Miguel Servet, Zaragoza, Spain; 4 CIBERCV (CIBER of Cardiovascular) Instituto de Salud Carlos III, Madrid, Spain; 5 Department of Medicine, University of Zaragoza, Zaragoza, Spain; 6 Unit of Cardiovascular and Nutritional Epidemiology, Institute of Environmental Medicine (IMM), Karolinska Institutet, Stockholm, Sweden; 7 CIBERESP (CIBER of Epidemiology and Public Health), Instituto de Salud Carlos III, Madrid, Spain; 8 IMDEA-Food Institute, Madrid, Spain

**Keywords:** Stilbenes, Flavonoids, Red wine, Subclinical coronary atherosclerosis, Coronary calcium, Cross-sectional cohort study

## Abstract

**Purpose:**

It is suggested that polyphenols back the cardiovascular protection offered by the Mediterranean diet. This study evaluates the association of specific types of dietary polyphenols with prevalent subclinical atherosclerosis in middle-aged subjects.

**Methods:**

Ultrasonography and TC were performed on 2318 men from the Aragon Workers Health Study, recruited between 2011 and 2014, to assess the presence of plaques in carotid and femoral arteries and coronary calcium. Polyphenol intake was assessed using a validated semi-quantitative 136-item food frequency questionnaire. The Phenol Explorer database was used to derive polyphenol class intake. Logistic and linear regressions were used to estimate the cross-sectional association of polyphenols intake with femoral and carotid subclinical atherosclerosis and coronary calcium.

**Results:**

A higher intake of flavonoids (third vs. first tertile) was associated with a lower risk of both carotid (OR 0.80: CI 95% 0.62–1.02; *P* trend 0.094) and femoral (0.62: 0.48–0.80, *P* trend < 0.001) subclinical atherosclerosis. A higher intake of stilbenes was associated with a lower risk of femoral subclinical atherosclerosis (0.62: 0.46–0.83; *P* trend 0.009) and positive coronary calcium (0.75: 0.55–1.03; *P* trend 0.131). A higher intake of tyrosols was also associated with a lower risk of positive coronary calcium (0.80: 0.62–1.03; *P* trend 0.111). The associations remained similar when adjusted for blood lipids and blood pressure.

**Conclusion:**

Dietary flavonoids, stilbenes, and tyrosols, whose main sources are red wine and virgin olive oil, are associated with lower prevalence of subclinical atherosclerosis in middle-aged subjects.

**Supplementary Information:**

The online version contains supplementary material available at 10.1007/s00394-022-02823-0.

## Background

Polyphenolic compounds are secondary plant metabolites found in a variety of plant-derived foods, including fruits, nuts, tea, cocoa products, coffee, vegetables, olive oil, soy products, as well as red wine [1]. Polyphenols comprise flavonoids (such as flavan-3-ols, flavonols, anthocyanins, flavanones, flavones, and isoflavones) and phenolic acids, stilbenes, lignans, and other minor polyphenols. These compounds are intensively studied for their potential health benefits in chronic diseases, such as cardiovascular diseases (CVD). High dietary polyphenol intake, especially flavonoids, has consistently been associated with a reduced incidence of cardiovascular events [2–5].

Protective effects of polyphenols against inflammatory and oxidative processes, and endothelial dysfunction, which are implicated in the atherosclerosis development, have also been evidenced [6–9]. In a sub-study of 1139 high-risk participants, which was carried out within the PREDIMED trial, a higher polyphenol exposure measured as urinary polyphenol excretion was associated with lower levels of inflammatory biomarkers, suggesting a dose-dependent anti-inflammatory effect of polyphenols. High polyphenol intake also improved blood pressure and the lipid profile [9].

Atherosclerosis, which underlies CVD, is a complex process in which fat, inflammation cells, scar tissue, and deposits of calcium accumulate within the walls of the arteries [10]. The presence of calcium in the coronary arteries is an indicator of subclinical atherosclerotic disease and a marker of coronary damage [11], as well as, a strong and independent predictor of future coronary heart disease [12, 13]. Also, the presence of atherosclerotic plaques in peripheral arteries is considered a valuable evaluation of subclinical atherosclerosis [14]. Association of atherosclerosis with risk factors may be stronger in femoral arteries than carotid or coronary arteries, at least in the middle-aged adult [14].

To date, however, no epidemiological study has yet evaluated the direct association of dietary polyphenol intake with subclinical atherosclerosis, a missing step from their influence in risk factors and pathogenic mechanisms toward clinical disease. The aim of this study is to examine the association of specific types of dietary polyphenols with plaques in the carotid and femoral arteries and coronary calcium in middle-aged asymptomatic subjects with a low prevalence of clinical comorbidities.

## Methods

### Study design and population

The Aragon Workers´ Health Study (AWHS) is a prospective cohort whose design and methodology has been described in detail elsewhere [14, 15]. Study participants are middle-aged workers, of the Opel Spain automobile assembly plant recruited during an annual physical examination in 2009–2010 (participation rate 95.6%). Between January 2011 and December 2014, all participants aged 39–59 (34% of the initial sample) and free of CVD at baseline were invited to undergo noninvasive subclinical atherosclerosis imaging as well as questionnaires on cardiovascular and lifestyle factors. The data used for this study were cross-sectional.

The study was approved by the Clinical Research Ethics Committee of Aragon (CEICA). All participants provided written informed consent.

### Dietary polyphenol intake

Dietary polyphenol intake was assessed at baseline using a 136-item FFQ previously validated and repeatedly re-evaluated in Spain [16–18]. The FFQ grouped the foods into the following categories: fruits (fresh, dried, jam, in the yogurt), vegetables, legumes, oils (olive, virgin olive, sunflower and corn), grains (rice and pasta), breads (white and whole), artisanal (homemade) and industrialized pastries, juices (natural, packaged), breakfast cereals, chocolate and cocoa products, coffee and tea beverages, and alcoholic drinks (wine, beer, spirits). The frequency of food consumption was collected by nine categories (ranging from never or almost never to more than six servings per day). A standard portion size for each food item was also included.

For the evaluation of the polyphenols intake, foods with only traces or without polyphenols (dairy, eggs, meat, fish, butter, margarine, lard, carbonated drinks, water, soups and creams, mayonnaise, salt, sugar, saccharin) were excluded [19].

Data on the polyphenol content in foods were obtained from the Phenol Explorer database (www.phenolexplorer.eu). For three specific foods not identified in Phenol Explorer (leek, thistle, and honey) we used the United States Department of Agriculture (USDA) database (https://www.ars.usda.gov/nutrientdata). Polyphenol content was analyzed by chromatography after hydrolysis and expressed as aglycones.

Polyphenol content from recipes and processed foods was calculated based on their separated ingredients. For raw foods, the Retention Factor (RF) was applied as a way to compensate for losses or gains in nutrients during food processing. A RF, considering the domestic cooking practices (i.e., boiling, steaming, frying, microwaving) [20] and processing, was applied for cooked and processed foods [19]. For this purpose, RF was considered when the polyphenol content was only available in the Phenol Explorer in the raw form of the food.

Individual polyphenol intake from each food was calculated by multiplying the individual polyphenol content by the daily consumption of each food. The total dietary polyphenol intake was calculated as the sum of all individual polyphenol intakes from all food sources reported by the FFQ. We evaluated total polyphenols and the following types: flavonoids, phenolic acids, stilbenes, lignans, and other polyphenols. Total intake and types of polyphenols were adjusted for total energy intake using the residual method [21].

### Subclinical atherosclerosis imaging

The presence of plaques in carotid and femoral arteries was determined using an ultrasound system IU22 Philips (Philips Healthcare, Bothell, Washington). Ultrasound images were acquired with linear high-frequency 2-dimensional probes (Philips Transducer L9-3, Philips Healthcare), using the Bioimage Study protocol for the carotid arteries [22] and a protocol that was specifically designed for the femoral arteries [23]. Inspection sweeps were obtained on the right and left side of the carotid (common, internal, external, and bulb) and femoral territories. The presence of a plaque was defined as a focal structure protruding ≥ 0.5 mm into the lumen artery or reaching a thickness ≥ 50% of the surrounding intima. All measurements were analyzed using electrocardiogram (ECG)-gated frames and obtained at the end of the diastole (R-wave)[24].

Coronary calcium was obtained with a multi-detector-row CT scanner (Mx 8000 IDT 16, Philips Medical Systems, Best, the Netherlands) using a low-dose, prospectively ECG-triggered, and a high-pitch spiral acquisition protocol. Coronary calcium was quantified with calcium scoring software (Workspace CT viewer, Philips Medical Systems) that follows the Agatston method [25]. Agatston’s method is a summed score obtained from all coronary calcified lesions, accounting for both, the total area as well as the maximum density of coronary calcium. A high coronary artery calcium score (CACS) is a strong indicator of extensive disease with a significant amount of calcium deposits. CACS is the reference standard and the most commonly used coronary artery calcium score in clinical practice [25]. Having a CACS > 0 represents the presence of calcium and has been associated with increases in coronary heart disease rates [26].

Imaging of subclinical atherosclerosis was performed at the AWHS Clinic located at the Hospital Universitario Miguel Servet in Zaragoza, Spain [14, 15].

### Additional data collection

Information on demographic characteristics was self-reported and included age, sex, marital status (married, not married), and educational level (middle school, high school, professional training, and college). Study participants undergo a standardized physical exam, including weight (kg), height (cm), waist circumference, blood pressure (BP), medical history, and the current use of medication. Hypertension was defined if use of antihypertensive medication was self-reported, or the reported systolic blood pressure was ≥ 131 mmHg or the diastolic ≥ 81 mmHg. Body mass index (BMI) (kg/m^2^) was calculated by dividing weight by height squared.

Each participant also provided a sample of blood and urine after overnight fasting (> 8 h) for laboratory analyses and for biobanking. Total cholesterol, high-density lipoprotein cholesterol (HDL-c), triglycerides, and fasting serum glucose concentrations were determined by enzyme analysis using the ILAB 650 analyzer from Instrumentation Laboratory (Bedford, MA, USA). Low-density lipoprotein cholesterol (LDL-c) was calculated using the Friedewald formula when the triglyceride levels were < 400 mg/dl [27]. Hypercholesterolemia was considered when use of lipid-lowering medication was reported or having total cholesterol ≥ 200 mg/dL.

Lifestyle factors including physical activity smoking, and sleep duration (both on weekdays and weekends) were obtained by questionnaires. The leisure-time physical activities and time spent in sedentary activities (including sleep duration) were assessed using a formerly validated questionnaire, i.e., the Health Professionals' Follow-up physical activity questionnaire [28]. Participants were asked about the time devoted to 17 different activities during the preceding year, and leisure-time physical activity was expressed in metabolic equivalents (METs)-h/week. Smoking habits were categorized as current smoking if the participant reported having smoked in the last year, former smoking if the participant had smoked at least 50 cigarettes in his lifetime, but not in the last year, and never smoking.

Physicians and nurses collecting these data underwent specific training and standardization programs organized by the study investigators. Compliance with study procedures was routinely monitored and deviations were corrected. The study conforms to the ISO9001-2008 quality standard. All study procedures are described elsewhere [15].

### Statistical analysis

Participants were categorized into tertiles of daily mg of total polyphenols intake, as well as into tertiles of each individual polyphenol intake, after adjusting for total energy intake using the residual method [21]. Spearman correlations were calculated between intakes of the different polyphenols. The contribution of the main food groups to polyphenol intake was also estimated.

We performed a standard binary logistic regression to estimate the odds ratio (OR) and corresponding 95% confidence intervals (CI) for the presence of at least one plaque in the carotid and femoral arteries as well as for having a positive coronary calcium score (CACS > 0 *vs*. CACS = 0) associated to total and per class dietary polyphenol intake. By linear regression, we also evaluated the mean difference of the CACS as log-transformed continuous score (log(CACS + 1)) according to tertiles of energy-adjusted dietary polyphenols intake. To calculate the P for trend, means of the polyphenol intakes in each tertile were used and treated as a continuous variable in the model. The ORs for the presence of plaques and positive CACS were also calculated using the polyphenols intake (mg/day) as a continuous variable.

We built three models with progressive adjustment for covariates that can operate as confounders [29]. Model 1 was adjusted for age (continuous, years) and total energy intake (Kcal/d); model 2 was further adjusted for marital status (married, not married), education (middle school, high school, professional training, and college), smoking (never, former, and current smoker), physical activity (in MET-h/wk.), BMI (< 25, 25 to < 30, ≥ 30 kg/m^2^), time spent sleeping during the weekdays (number of hours of sleep, continuous), time spent sleeping during the weekend (number of hours of sleep, continuous), alcohol consumption (g/d), total dietary fiber (g/day), and use of diabetes medication (yes, no); model 2 was considered the main model to assess the associations of polyphenol intake with subclinical atherosclerosis; and additionally, a model 3 was further adjusted for cardio-metabolic risk factors, which can potentially act as mediators, i.e., LDL cholesterol in blood (mg/dL), HDL cholesterol in blood (mg/dL), and systolic and diastolic blood pressure (mmHg). Because flavonoids and stilbenes were the polyphenols more correlated, we presented and additional model 4, mutually adjusting for flavonoids and stilbenes when appropriate.

To obtain unbiased estimates, missing values (< 1% of total values) were imputed using multiple imputation with chained equations with ten predictors (age, marital status, physical activity, total dietary fiber, total energy intake, alcohol consumption, LDL cholesterol, HDL cholesterol, systolic and diastolic blood pressure) and ten imputations [30, 31]. The validity of the imputation was checked by comparing the results obtained with the effects resulting from the analyses of the data with complete information for all variables. The complete case sensitivity analyses led to very similar results (data not shown).

We also performed interaction analysis between polyphenols and age, BMI, and smoking. P for interaction was obtained using the likelihood ratio test of the models with and without the interaction term. Finally, sensitivity analyses were performed after excluding those with prevalent diabetes (4.2%).

Participants’ characteristics were compared across the three categories of total polyphenol intake, using counts and percentages for categorical variables and means and standard deviations for continuous variables. *P* value estimates were based on one-way ANOVA for continuous variables and Pearson χ^2^ test for categorical variables. For variables with imputed values, we used the *P* values from ordered logistic regression models.

The software used for statistical analysis was STATA/SE version 16.0 (Stata Corporation, Inc., Collage Station, TX, USA). All tests were two sided with the level of significance set at 0.05.

## Results

Among the 2586 workers recruited into the AWHS imaging study, we excluded a minority of women (*n* = 132), and those with an extreme total energy intake (< 600 or > 4200 kcal) (n = 136), resulting in 2318 participants. Among them, plaques measurements in carotid and femoral were only available in 2183 and 2187 participants, respectively, and CACS measurements were only available for 1876 (Supplemental Fig. 1).

Total polyphenol intake ranged from 562 (± 173) in the lowest tertile to 1314 (± 326) mg/d in the highest. The class of polyphenols consumed the most (50% of the total intake) was flavonoids (546 ± 264 mg/d), and those consumed the least were stilbenes (4.5 ± 6.1 mg/d) and lignans (3.0 ± 1.3 mg/d), both together representing less than 1% of the total polyphenol intake (Table 1).

**Table 1:** Characteristics of the study participants according to quartiles of energy-adjusted total polyphenols intake, the AWHS study (*N* = 2318)

	Energy-adjusted total polyphenols intake (mg/d)*				
	Tertile 1	Tertile 2	Tertile 3	*P* value	
*n*	773	773	772		
Total polyphenols (mg/d)	754 (197)	1055 (186)	1501 (332)	< 0.001	
Flavonoids (mg/d)	357 (137)	512 (162)	769 (279)	< 0.001	
Phenolic acids (mg/d)	349 (130)	478 (137)	630 (191)	< 0.001	
Stilbenes (mg/d)	1.9 (3.09)	4.09 (4.97)	7.43 (7.79)	< 0.001	
Lignans (mg/d)	2.55 (1.12)	2.35 (1.14)	2.5 (1.27)	< 0.001	
Tyrosols (mg/d)	30.8 (26.6)	42.7 (32.4)	72.6 (61.9)	< 0.001	
Alkylphenols (mg/d)	7.8 (17.1)	10.5 (19.4)	12 (23.4)	< 0.001	
Total energy intake (kcal)	2865 (610)	2735 (606)	2837 (609)	< 0.001	
Age (years)	50.4 (4)	50.8 (3.9)	51.4 (3.7)	< 0.001	
Married (%)	81	86.7	85.1	0.007	
Education (%)				0.019	
Middle school	54.6	46.6	48.7		
High school	9.11	13.1	9.18		
Professional training	32.5	33.5	35.2		
College	3.76	6.77	6.92		
Smoking (%)				< 0.001	
Never	23.2	24.5	23.1		
Former	36.3	30.9	27.4		
Current	40.5	44.7	49.5		
Physical activity (MET-h/week)	30.3 (21.3)	31.2 (22.5)	34.4 (24.1)	< 0.001	
Body mass index (%)				0.139	
< 25 kg/m^2^	20.1	16.8	19.8		
25 to < 30 kg/m^2^	56.9	56.4	58.2		
≥ 30 kg/m^2^	23.0	26.8	22.0		
Sleep duration (hours)					
During the weekdays	6.31 (.95)	6.37 (.93)	6.26 (.98)	0.0887	
During the weekend	7.28 (1.19)	7.38 (1.1)	7.25 (1.19)	0.0894	
Alcohol consumption (g/d)	16.2 (17.1)	19.3 (17.8)	26.3 (21.7)	< 0.001	
Total fiber intake (g/d)	22.8 (6.4)	24.5 (7.4)	27.3 (8.1)	< 0.001	
LDL cholesterol in blood (mg/dL)	136 (35)	137 (31)	138 (33)	0.4237	
HDL cholesterol in blood (mg/dL)	51.8 (11.3)	53.1 (11.3)	54.1 (11.5)	< 0.001	
Blood pressure (mmHg)	125 (14)	126 (14)	125 (14)		
Systolic	82.8 (9.7)	83.1 (9.5)	82.4 (9.2)	0.3342	
Diastolic	6.31 (.95)	6.37 (.93)	6.26 (.98)	0.3785	
Diabetes medication use (%)	4.51	3.89	4.16	0.738	
Hypertension	64.8	68.6	65.7	0.263	
Hypercholesterolemia	74.9	77	77.6	0.427	
Obesity	23	26.8	22	0.069	
Main food groups intake (g/day)					
Fruit	239 (153)	298 (165)	364 (183)	< 0.001	
Vegetables	272 (110)	318 (127)	364 (136)	< 0.001	
Legumes	33.3 (12.1)	32.7 (11.7)	33.9 (14.1)	0.187	
Virgin olive oil	18.6 (20.6)	20.1 (20.2)	21.6 (20.6)	0.013	
Coffee	86.0 (57)	121 (65)	149 (81)	< 0.001	
Nuts	2.44 (3.98)	3.54 (5.65)	5.57 (8.37)	< 0.001	
Chocolate	2.09 (4.21)	3.55 (5.59)	7.02 (10.38)	< 0.001	
Red wine	33.5 (58.9)	76 (95.7)	139.8 (150.2)	< 0.001	

Continuous variables are presented as mean ± standard deviation and categorical variables as percentage. *P* value estimates are based on one-way ANOVA for variables expressed as mean (standard deviation) or Pearson’s χ^2^ test for variables expressed as percentages. For variables with imputed values, we used the *P* values from ordered logistic regression models

*AWHS* Aragon Workers' Health Study

*Energy-adjusted by the residual method

All participants were Caucasian males with a mean age of 51 ± 4 years. Among them, 4% were diabetic, 66% were hypertensive, 76% had hypercholesterolemia, and 24% were obese. Compared to those in the lowest tertile of total polyphenol intake, those in the highest were on average 1-year older, had more education, were more frequently current smokers, performed more physical activity, had a higher consumption of alcohol, and dietary fiber, as well as higher levels of HDL cholesterol (Table 1). Furthermore, those with the highest total polyphenol intake consumed more fruit, vegetables, virgin olive oil, coffee, nuts, chocolate, and red wine (Table 1).

The contributions of different foods to the total and types of polyphenol intake are shown in Table 2. Coffee, followed by red wine were the major contributors to the total polyphenol intake. Apples and pears, red wine, cherries/ plums, and chocolate were the major contributors to flavonoids; coffee to phenolic acids; red wine to stilbenes; white bread to lignans; virgin olive oil to tyrosols; and whole bread coffee to alkylphenols.

**Table 2:** Main food contributors to polyphenols (% of contribution)

Food	Percentage of contribution
Total polyphenols	
Coffee	25.4
Red wine	9.12
Apple/Pear	8.49
Cherries and plums	6.76
Chocolate	5.92
Nuts	3.45
Legumes	3.36
Flavonoids	
Apple/Pear	14.3
Red wine	14.0
Cherries and plums	11.1
Chocolate	10.7
Legumes	5.56
Phenolic acids	
Coffee	51.6
Potatoes	5.07
Apple/Pear	3.95
Cherries and plums	3.24
Red wine	3.11
Stilbenes	
Red wine	48.8
Legumes	9.52
Nuts	2.12
Chocolate	2.05
Lignans	
White bread	52.9
Nuts	10.5
*Gazpacho*^*1*^	8.82
Virgin olive oil	8.37
Whole bread	8.08
Tyrosols	
Virgin olive oil	18.1
*Gazpacho*^*1*^	10.4
Red wine	8.82
Alkylphenols	
Whole bread	16.2
Coffee	11.8

^1^*Gazpacho* is a cold mainly made of tomato, bell pepper, cucumber, olive oil, and garlic

Spearman’s correlations between the different polyphenol groups were mild to moderate; the higher correlation occurred between flavonoids and stilbenes (*ρ* 0.54) (Supplemental Table 1).

Subclinical atherosclerosis was found in 72% of participants. Atherosclerosis was most common as femoral plaques (57%), followed by coronary calcification (40%) and carotid plaques (38%). A tendency to a lower risk of plaques in the carotid arteries (-20%) was only observed in those subjects with a highest intake of flavonoids when compared to those with lowest consumption (Model 2, OR 0.80: CI 95% 0.62–1.02; *P* trend 0.094) (Table 3). This association was maintained after adjusting for blood lipids and blood pressure (Model 3) and was minimally diluted when adjusting for stilbenes (Model 4). A similar lower risk of plaques in the femoral arteries (− 38%) was observed in those subjects with a higher intake of flavonoids (Model 2 OR 0.62: 0.48–0.80, *P* trend < 0.001) but also in those with higher stilbenes intake (Model 2 OR 0.62: 0.46–0.83; *P* trend 0.009) compared to those with lower consumption (Table 4). Likewise, the risk of plaques in the femoral arteries was reduced by a 4% (OR 0.94; CI 95% 0.90–0.98) for each 100 mg/day intake of flavonoids; and a 3% (OR 0.97; CI 95% 0.95–0.99) decreased risk of plaques in the femoral arteries for each 1 mg/day intake of stilbenes (not shown in tables).

**Table 3:** Odds ratio (OR) and 95% confidence intervals (CI) for the risk of the presence of at least one plaque in the carotid arteries according to tertiles of energy-adjusted dietary polyphenols intake (*n* = 2183)

	Tertiles of energy-adjusted dietary polyphenols intake (mg/d)			
	Tertile 1	Tertile 2	Tertile 3	*P* trend
Total polyphenols				
Prevalent cases/*n*	268/721	270/732	296/730	
Model 1. OR (95%CI)	Ref.	0.93 (0.75, 1.15)	1.02 (0.82, 1.27)	0.793
Model 2. OR (95%CI)	Ref.	0.95 (0.76, 1.19)	1.02 (0.80, 1.29)	0.875
Model 3. OR (95%CI)	Ref.	0.95 (0.75, 1.20)	1.03 (0.81, 1.32)	0.776
Flavonoids				
Prevalent cases/*n*	291/724	267/730	276/729	
Model 1. OR (95%CI)	Ref.	0.77 (0.62, 0.96)	0.80 (0.64, 0.99)	0.064
Model 2. OR (95%CI)	Ref.	0.79 (0.63, 1.00)	0.80 (0.62, 1.02)	0.094
Model 3. OR (95%CI)	Ref.	0.77 (0.61, 0.97)	0.81 (0.63, 1.03)	0.126
Model 4 OR (95%CI)	Ref.	0.78 (0.61, 0.99)	0.84 (0.64, 1.10)	0.263
Phenolic acids				
Prevalent cases/*n*	253/725	280/730	301/728	
Model 1. OR (95%CI)	Ref.	1.14 (0.91, 1.42)	1.22 (0.98, 1.52)	0.074
Model 2. OR (95%CI)	Ref.	1.14 (0.91, 1.42)	1.18 (0.94, 1.48)	0.153
Model 3. OR (95%CI)	Ref.	1.15 (0.91, 1.44)	1.20 (0.95, 1.50)	0.130
Stilbenes				
Prevalent cases/*n*	265/729	264/723	305/731	
Model 1. OR (95%CI)	Ref.	0.93 (0.74, 1.18)	1.07 (0.86, 1.34)	0.311
Model 2. OR (95%CI)	Ref.	0.92 (0.72, 1.17)	0.89 (0.67, 1.18)	0.536
Model 3. OR (95%CI)	Ref.	0.94 (0.73, 1.20)	0.93 (0.70, 1.25)	0.758
Model 4. OR (95%CI)	Ref.	0.94 (0.74, 1.21)	0.96 (0.71, 1.29)	0.889
Lignans				
Prevalent cases/*n*	264/730	294/725	276/728	
Model 1. OR (95%CI)	Ref.	1.17 (0.94, 1.45)	1.05 (0.84, 1.30)	0.741
Model 2. OR (95%CI)	Ref.	1.18 (0.95, 1.48)	1.07 (0.85, 1.34)	0.609
Model 3. OR (95%CI)	Ref.	1.18 (0.94, 1.47)	1.07 (0.85, 1.35)	0.600
Tyrosols				
Prevalent cases/*n*	273/730	260/723	301/730	
Model 1. OR (95%CI)	Ref.	0.87 (0.70, 1.09)	1.02 (0.82, 1.27)	0.629
Model 2. OR (95%CI)	Ref.	0.86 (0.69, 1.09)	1.00 (0.80, 1.26)	0.747
Model 3. OR (95%CI)	Ref.	0.88 (0.70, 1.11)	1.03 (0.82, 1.30)	0.590
Alkylphenols				
Prevalent cases/*n*	394/720	289/738	251/725	
Model 1. OR (95%CI)	Ref.	0.98 (0.78, 1.23)	0.85 (0.68, 1.07)	0.117
Model 2. OR (95%CI)	Ref.	1.05 (0.83, 1.32)	0.97 (0.76, 1.23)	0.598
Model 3. OR (95%CI)	Ref.	1.06 (0.83, 1.33)	1.00 (0.79, 1.28)	0.849

Model 1: Logistic regression model adjusted for age and total energy intake. Model 2: As in Model 1 and additionally adjusted for marital status, education, smoking, physical activity, sleep duration during weekdays and during the weekend, alcohol consumption, total fiber intake, body mass index, and diabetes medication use. Model 3: As in Model 2 and additionally adjusted for LDL and HDL cholesterol and systolic and diastolic blood pressure. Model 4: As in Model 3 and flavonoids and stilbenes were mutually adjusted

*CI* confidence interval, *OR* odds ratio

**Table 4:** Odds ratio (OR) and 95% confidence intervals (CI) for the risk of the presence of at least one plaque in the femoral arteries according to tertiles of energy-adjusted dietary polyphenols intake (*n* = 2187)

	Tertiles of energy-adjusted dietary polyphenols intake (mg/d)			
	Tertile 1	Tertile 2	Tertile 3	*P* trend
Total polyphenols				
Prevalent cases/*n*	426/718	406/731	404/738	
Model 1. OR (95%CI)	Ref.	0.82 (0.66, 1.01)	0.74 (0.59, 0.91)	0.006
Model 2. OR (95%CI)	Ref.	0.87 (0.69, 1.10)	0.82 (0.64, 1.05)	0.121
Model 3. OR (95%CI)	Ref.	0.87 (0.69, 1.10)	0.84 (0.65, 1.08)	0.170
Flavonoids				
Prevalent cases/*n*	458/729	400/716	378/742	
Model 1. OR (95%CI)	Ref.	069 (0.55, 0.86)	0.53 (0.43, 0.66)	< 0.001
Model 2. OR (95%CI)	Ref.	0.79 (0.62, 1.00)	0.62 (0.48, 0.80)	< 0.001
Model 3. OR (95%CI)	Ref.	0.76 (0.60, 0.97)	0.63 (0.49, 0.81)	< 0.001
Model 4. OR (95%CI)	Ref.	0.77 (0.61, 0.99)	0.66 (0.50, 0.87)	0.003
Phenolic acids				
Prevalent cases/*n*	487/721	410/735	439/731	
Model 1. OR (95%CI)	Ref.	1.07 (0.86, 1.32)	1.22 (0.98, 1.50)	0.070
Model 2. OR (95%CI)	Ref.	1.06 (0.84, 1.33)	1.16 (0.92, 1.46)	0.195
Model 3. OR (95%CI)	Ref.	1.07 (0.85, 1.35)	1.18 (0.93, 1.49)	0.162
Stilbenes				
Prevalent cases/*n*	434/725	387/729	415/733	
Model 1. OR (95%CI)	Ref.	0.71 (0.57, 0.89)	0.74 (0.59, 0.92)	0.073
Model 2. OR (95%CI)	Ref.	0.73 (0.57, 0.94)	0.62 (0.46, 0.83)	0.009
Model 3. OR (95%CI)	Ref.	0.74 (0.58, 0.96)	0.66 (0.49, 0.90)	0.035
Model 4. OR (95%CI)	Ref.	0.77 (0.59, 0.99)	0.73 (0.53, 1.00)	0.166
Lignans				
Prevalent cases/*n*	413 /725	402/729	421/733	
Model 1. OR (95%CI)	Ref.	0.89 (0.72, 1.10)	1.00 (0.81, 1.23)	0.958
Model 2. OR (95%CI)	Ref.	0.84 (0.67, 1.06)	0.99 (0.79, 1.25)	0.962
Model 3. OR (95%CI)	Ref.	0.83 (0.66, 1.04)	0.99 (0.78, 1.25)	0.978
Tyrosols				
Prevalent cases/*n*	419/725	404/734	413/728	
Model 1. OR (95%CI)	Ref.	0.86 (0.69, 1.07)	0.85 (0.69, 1.05)	0.184
Model 2. OR (95%CI)	Ref.	0.90 (0.71, 1.13)	0.90 (0.71, 1.14)	0.458
Model 3. OR (95%CI)	Ref.	0.90 (0.71, 1.13)	0.91 (0.71, 1.16)	0.511
Alkylphenols				
Prevalent cases/*n*	428/726	419/730	389/731	
Model 1. OR (95%CI)	Ref.	0.94 (0.75, 1.18)	0.84 (0.68, 1.05)	0.132
Model 2. OR (95%CI)	Ref.	0.93 (0.74, 1.19)	1.07 (0.84, 1.37)	0.333
Model 3. OR (95%CI)	Ref.	0.93 (0.73, 1.18)	1.14 (0.89, 1.46)	0.129

Model 1: Logistic regression model adjusted for age and total energy intake. Model 2: As in Model 1 and additionally adjusted for marital status, education, smoking, physical activity, sleep duration during weekdays and during the weekend, alcohol consumption, total fiber intake, body mass index, and diabetes medication use. Model 3: As in Model 2 and additionally adjusted for LDL and HDL cholesterol and systolic and diastolic blood pressure. Model 4: As in Model 3 and flavonoids and stilbenes were mutually adjusted

*CI* confidence interval, *OR* odds ratio

The participants CACS mean was 122 ± 282. In this sample, those participants with the highest intake of stilbenes reduced their risk of having a positive CACS by 25% (OR 0.75; CI 95% 0.55–1.03), and those with the highest intake of tyrosols the reduction was 20% (OR 0.80; CI 95% 0.62–1.03) (Table 5). Also, when assessing the CACS as continuous, those with higher intake of stilbenes divided their CACS by 1.46, on average, with respect to those with the lowest tertile of intake (Model 2 difference of log-transformed CACS -0.41: − 0.69 to − 0.14; *P* trend 0.019) (Supplemental Table 2).

**Table 5:** Odds ratio (OR) and 95% confidence intervals (CI) for the risk of a positive coronary calcium Agatston Score (CACS > 0) according to tertiles of energy-adjusted dietary polyphenols intake (*n* = 1876)

	Tertiles of energy-adjusted dietary polyphenols intake (mg/d)			
	Tertile 1	Tertile 2	Tertile 3	*P* trend
Total polyphenols				
Prevalent cases/*n*	239/611	254/638	254/627	
Model 1. OR (95%CI)	Ref.	0.97 (0.76, 1.22)	0.91 (0.72, 1.15)	0.415
Model 2. OR (95%CI)	Ref.	0.92 (0.72, 1.18)	0.84 (0.64, 1.09)	0.183
Model 3. OR (95%CI)	Ref.	0.92 (0.72, 1.18)	0.84 (0.64, 1.10)	0.206
Flavonoids				
Prevalent cases/*n*	244/622	251/627	252/627	
Model 1. OR (95%CI)	Ref.	0.93 (0.73, 1.18)	0.91 (0.72, 1.15)	0.441
Model 2. OR (95%CI)	Ref.	0.94 (0.73, 1.21)	0.88 (0.67, 1.15)	0.346
Model 3. OR (95%CI)	Ref.	0.93 (0.72, 1.19)	0.90 (0.68, 1.17)	0.438
Model 4. OR (95%CI)	Ref.	0.95 (0.74, 1.23)	0.96 (0.72, 1.28)	0.807
Phenolic acids				
Prevalent cases/*n*	239/626	243/614	265/636	
Model 1. OR (95%CI)	Ref.	1.02 (0.80, 1.29)	1.05 (0.84, 1.33)	0.652
Model 2. OR (95%CI)	Ref.	0.98 (0.77, 1.24)	0.99 (0.78, 1.26)	0.950
Model 3. OR (95%CI)	Ref.	0.97 (0.76, 1.24)	0.99 (0.78, 1.27)	0.956
Stilbenes				
Prevalent cases/*n*	237/622	237/617	273/637	
Model 1. OR (95%CI)	Ref.	0.90 (0.70, 1.15)	1.01 (0.80, 1.28)	0.652
Model 2. OR (95%CI)	Ref.	0.84 (0.64, 1.09)	0.75 (0.55, 1.03)	0.131
Model 3. OR (95%CI)	Ref.	0.86 (0.66, 1.13)	0.78 (0.57, 1.07)	0.180
Model 4. OR (95%CI)	Ref.	0.87 (0.66, 1.13)	0.79 (0.57, 1.10)	0.236
Lignans				
Prevalent cases/*n*	249/632	252/611	246/633	
Model 1. OR (95%CI)	Ref.	0.98 (0.78, 1.24)	0.92 (0.72, 1.16)	0.449
Model 2. OR (95%CI)	Ref.	0.97 (0.77, 1.24)	1.92 (0.73, 1.18)	0.522
Model 3. OR (95%CI)	Ref.	0.98 (0.77, 1.25)	0.91 (0.71, 1.16)	0.448
Tyrosols				
Prevalent cases/*n*	241/610	245/625	261/641	
Model 1. OR (95%CI)	Ref.	0.88 (0.69, 1.12)	0.85 (0.67, 1.08)	0.220
Model 2. OR (95%CI)	Ref.	0.86 (0.67, 1.10)	0.80 (0.62, 1.03)	0.111
Model 3. OR (95%CI)	Ref.	0.86 (0.67, 1.11)	0.82 (0.64, 1.06)	0.156
Alkylphenols				
Prevalent cases/*n*	260/648	245/626	242/626	
Model 1. OR (95%CI)	Ref.	1.02 (0.80, 1.31)	1.14 (0.90, 1.45)	0.241
Model 2. OR (95%CI)	Ref.	1.07 (0.84, 1.38)	1.31 (1.01, 1.70)	0.040
Model 3. OR (95%CI)	Ref.	1.08 (0.84, 1.39)	1.34 (1.03, 1.74)	0.027

Model 1: Logistic regression model adjusted for age and total energy intake. Model 2: As in Model 1 and additionally adjusted for marital status, education, smoking, physical activity, sleep duration during weekdays and during the weekend, alcohol consumption, total fiber intake, body mass index, and diabetes. Model 3: As in Model 2 and additionally adjusted for LDL and HDL cholesterol and systolic and diastolic blood pressure. Model 4: As in Model 3 and flavonoids and stilbenes were mutually adjusted

*CACS* Coronary Calcium Agatston Score, *CI* confidence interval, *OR* odds ratio

Similar results were obtained when models were adjusted for non-wine alcohol consumption. There was no evidence of interaction by age, BMI, or smoking. Also, after excluding participants with diabetes, the results and main conclusions remained (data not shown).

## Discussion

This is the first epidemiological evidence on the association between dietary polyphenol intake and subclinical atherosclerosis. In this sample of middle-aged male workers, among all types of polyphenols estimated from diet, flavonoids, stilbenes and tyrosols were those polyphenols more consistently associated with a lower prevalence of subclinical atherosclerosis. Dietary flavonoids (mainly from apples/ pears, red wine, cherries/ plums, and chocolate) were associated with lower prevalence of plaques in the carotid and femoral arteries, while stilbenes (largely from red wine) were associated with a lower prevalence of plaques in the femoral arteries and lower coronary calcium. Tyrosols also suggested having a protective effect against coronary calcium. These associations persisted after adjusting for blood lipids and blood pressure, known mediators of atherosclerosis.

A meta-analysis of 14 prospective cohort studies have shown that consumption of flavonoid-rich diets significantly decreased the risk of CVD [32]. A prior prospective cohort study of middle-aged Spanish adults found that, among different type of polyphenols, only dietary flavonoids were associated with a lower incidence of cardiovascular events [2]. In other Spanish cohort study of older adults at high cardiovascular risk, higher intakes of total polyphenols, lignans, flavonoids and hydroxybenzoic acids were associated with a lower risk of CVD [5] and subjects with high polyphenol intake, especially stilbenes and lignans, exhibited a reduced risk of overall mortality compared to lower intakes, but no significant associations for flavonoids or phenolic acids with all-cause mortality were found [5]. In this study, only flavonoids and stilbenes, whose main common source is red wine, were associated with lower prevalence of subclinical atherosclerosis.

Beyond the cardiovascular risk reduction already evidenced [33], light to moderate intake of red wine produces several beneficial effects on the vascular wall and blood cells and targeting all phases of the atherosclerotic process, from atherogenesis (functional disorder as flow-mediated dilatation, early plaque development, and growth) to vessel occlusion (thrombosis) [34]. Although it is known that ethanol favorably modifies the lipid pattern by decreasing total plasma cholesterol, in particular LDL, and by increasing HDL cholesterol, cardiovascular risk reduction attributed to wine is suggested to be linked largely to the effect of non-alcoholic components, mainly resveratrol, on the vascular wall and blood cells [35, 36].

In this study population, predominant source of both flavonoids and stilbenes was red wine and, formerly we also had demonstrated that moderate alcohol consumption was associated with lower prevalence of femoral artery subclinical atherosclerosis in this same cohort. Atherosclerosis was lower in ever-smokers who consumed between 2 g/d and 30 g/d with respect to those ever-smokers who were abstainers (OR 0.70; 95% CI 0.49–0.99; *P* < 0.05) [37]. Our results are adjusted for alcohol, and therefore, the effect of flavonoids and stilbenes is independent of the ethanol present in the wine.

Stilbenes are non-flavonoid polyphenols, characterized by the presence of a 1,2-diphenylethylene nucleus in their structure, and resveratrol and its derivatives are its main representatives [38]. Stilbenes are present mainly in red grapes, red wine, some kinds of tea, berries and peanuts, though their levels are very low in foods overall [39]. Notoriously, in this sample, while flavonoids represented close to 42% of the total dietary polyphenols intake, stilbenes represented less than 0.5%. However, it is known that the polyphenols that are the most common in the human diet are not necessarily the most active within the body, either because they have a lower intrinsic activity or because they are poorly absorbed from the intestine, highly metabolized, or rapidly eliminated [1]. Thus, stilbenes are among the most biologically active polyphenols contained in wine and, among them, resveratrol (trans-3,4′,5-trihydroxystilbene) has shown several benefits on the vascular system including anti-atherogenic, anti-inflammatory and anti-oxidative effects [40, 41]. Resveratrol has been found in urine samples of subjects who have drunk a glass of wine per week or three glasses per week after 3 or 5 days after last consumption, respectively [42]. Because resveratrol is mainly present in grape berry skins but not in flesh, and a major factor influencing its production is the fermentation time, white wine (which traditionally undergoes a shorter maceration time) contains only low amounts of resveratrol as compared with red wine [43]. While fruits were the major source of flavonoids in the Spanish population [39], the specific sources of variability in flavonoids intake in this study population were cacao, wine, and cherries/plums. This coincided with another study in the Spanish population [2].

Several plausible underlying biological mechanisms have been postulated to explain the beneficial effects of the polyphenols contained in red wine on the progression of atherosclerosis [40, 44]. Beneficial effects of resveratrol on vascular function go beyond its potential to inhibit the generation of oxidative stress/reactive oxygen species. Further effects of resveratrol in favor of the prevention of atherosclerosis include regulation of vasodilator and vasoconstrictor production, anti-inflammation, inhibition of modification of low-density lipoproteins, anti-platelet aggregation [40, 41, 45]. Resveratrol suppresses inflammation by inhibiting cyclooxygenase-1 and-2; lipoxygenases, epoxygenases and synthesis of prostaglandins and eicosanoids, and altering the nitric oxide generation [46]. Flavonoids also share many of these anti-atherogenic effects [47, 48].

Several limitations of our study must be recognized. First, differences between individuals in the absorption and metabolism of these plant bioactive compounds and the heterogeneity in their biological response [49] have not been taken into account. Likewise, although the FFQ provides an adequate assessment of an individual's usual diet [17] and some validation studies have shown that FFQs are reasonable tools for estimating polyphenols intake [50], because of its self-reported nature and the potential recall bias, potential inaccuracies in the dietary assessment may exist. Also, the estimation of polyphenol intake was performed using Phenol-Explore database with the exception of three specific foods (leek, thistle, and honey), for which it had to be used the United States Department of Agriculture (USDA) database (https://www.ars.usda.gov/nutrientdata). Consequently, we cannot rule out the existence of a certain level of information misclassification (although it would be a non-differential misclassification since the error would be unrelated to the presence of the outcome). Because polyphenol intake measurement using dietary questionnaires is challenging, biomarkers for polyphenol exposure would be very useful for validating these findings in future research.

Second, the cross-sectional design of our study prevents us from establishing a causal link between polyphenols intake and subclinical atherosclerosis. However, since the atherosclerosis is subclinical, reverse causation is highly unlikely. Third, the limited external validity of our findings should also be mentioned, as the cohort was not representative of the general population. However, there is no biological evidence by which these results found in male workers might not be extended to the general population. And finally, despite adjusting for a wide range of potential confounders, we cannot rule out residual confounding.

This study notably presents important strengths, such as its novelty, the quality of the methodology used to collect clinical data and to quantify plaques in different territories and coronary calcium. These measurements capture information about the atherosclerosis distribution and have strong published support of their value for clinical risk prediction. Also, the detailed data collection for confounders, including accurate measurements of blood pressure and serum lipids, helps reduce confounding.

As a conclusion, in this study of middle-age Spanish working men, we found that those consuming the highest amount of dietary flavonoids and stilbenes have lower prevalence of subclinical atheroma plaques and coronary calcium. Thus, the consumption of foods high in these compounds could prevent cardiovascular risk from very early stages.

## Supplementary Information

Below is the link to the electronic supplementary material.

Supplementary file1 (PDF 107 KB)

Supplementary file2 (PDF 36 KB)

## Data Availability

The datasets used and/or analyzed during the current study are available from the corresponding author on reasonable request.

## Abbreviations

AWHS: Aragon Workers´ Health Study
CVD: Cardiovascular disease
CI: Confidence intervals
CACS: Coronary Artery Calcium Score
FFQ: Food Frequency Questionnaire
OR: Odds ratio
RF: Retention factor
