# Correction to: The intake of flavonoids, stilbenes, and tyrosols, mainly consumed through red wine and virgin olive oil, is associated with lower carotid and femoral subclinical atherosclerosis and coronary calcium

**DOI:** 10.1007/s00394-022-02948-2

**Published:** 2022-07-12

**Authors:** Henry Montero-Salazar, Raquel de Deus Mendonça, Martín Laclaustra, Belén Moreno-Franco, Agneta Åkesson, Pilar Guallar-Castillón, Carolina Donat-Vargas

**Affiliations:** 1 Department of Preventive Medicine and Public Health and Microbiology, Universidad Autónoma de Madrid, CEI UAM+CSIC+ IdiPaz, Madrid, Spain; 2 Department of Clinical and Social Nutrition. School of Nutrition, Universidade Federal de Ouro Preto, Ouro Preto, Brasil; 3 Insituto de Investigación Sanitaria (IIS) Aragón, Hospital Universitario Miguel Servet, Zaragoza, Spain; 4 CIBERCV (CIBER of Cardiovascular) Instituto de Salud Carlos III, Madrid, Spain; 5 Department of Medicine, University of Zaragoza, Zaragoza, Spain; 6 Unit of Cardiovascular and Nutritional Epidemiology, Institute of Environmental Medicine (IMM), Karolinska Institutet, Stockholm, Sweden; 7 CIBERESP (CIBER of Epidemiology and Public Health), Instituto de Salud Carlos III, Madrid, Spain; 8 IMDEA-Food Institute, Madrid, Spain

## Correction to: European Journal of Nutrition 10.1007/s00394-022-02823-0

The original version of this article unfortunately contained a mistake. The author’s name Henry Montero-Salazar was incorrectly written as Henry Montero Salazar.

