# Supplementary material for: The intake of flavonoids, stilbenes, and tyrosols, mainly consumed through red wine and virgin olive oil, is associated with lower carotid and femoral subclinical atherosclerosis and coronary calcium
Source: Eur J Nutr. 2022 Mar 7;61(5):2697–709. doi: 10.1007/s00394-022-02823-0 (PMC9279214; doi:10.1007/s00394-022-02823-0)
Supplement: Supplementary file 1 — Supplementary file1 (PDF 107 KB) [file 394_2022_2823_MOESM1_ESM.pdf]

**Supplemental Table 1.** Spearman correlations between the different classes of polyphenols

| | Flavonoids | Phenolic acids | Stilbenes | Lignans | Tyrosols | Alkylphenols |
|---|---|---|---|---|---|---|
| Flavonoids | 1.000 | | | | | |
| Phenolic acids | 0.281 | 1.000 | | | | |
| Stilbenes | 0.546 | 0.167 | 1.000 | | | |
| Lignans | 0.186 | 0.130 | 0.135 | 1.000 | | |
| Tyrosols | 0.339 | 0.406 | 0.329 | 0.292 | 1.000 | |
| Alkylphenols | 0.107 | 0.100 | 0.003 | 0.094 | -0.014 | 1.000 |

**Supplemental Table 2.** Multivariable mean difference and 95% confidence intervals (CI) of the **Coronary Calcium Agatston Score (CACS)*** according to tertiles of energy-adjusted dietary polyphenols intake (n=1,876).

| | **Tertiles of energy-adjusted dietary polyphenols intake (mg/d)** | | | |
|---|---|---|---|---|
| | Tertile 1 | Tertile 2 | Tertile 3 | *P* trend |
| **Total polyphenols** | | | | |
| Model 1. β (95%CI) | Ref. | 0.01 (-0.21, 0.22) | -0.08 (-0.29, 0.14) | 0.481 |
| Model 2. β (95%CI) | Ref. | -0.02 (-0.23, 0.12) | -0.12 (-0.35, 0.12) | 0.316 |
| Model 3. β (95%CI) | Ref. | -0.03 (-0.24, 0.19) | -0.11 (-0.35, 0.12) | 0.344 |
| **Flavonoids** | | | | |
| Model 1. β (95%CI) | Ref. | -0.05 (-0.27, 0.16) | -0.05 (-0.26, 0.17) | 0.698 |
| Model 2. β (95%CI) | Ref. | 0.02 (-0.24, 0.20) | -0.02 (-0.26, 0.22) | 0.880 |
| Model 3. β (95%CI) | Ref. | -0.03 (-0.25, 0.19) | -0.00 (-0.24, 0.23) | 0.995 |
| Model 4. β (95%CI) | Ref. | 0.02 (-0.21, 0.24) | 0.13 (-0.12, 0.38) | 0.298 |
| **Phenolic acids** | | | | |
| Model 1. β (95%CI) | Ref. | 0.10 (-0.12, 0.31) | 0.03 (-0.19, 0.24) | 0.857 |
| Model 2. β (95%CI) | Ref. | 0.05 (-0.16, 0.26) | -0.04 (-0.25, 0.18) | 0.703 |
| Model 3. β (95%CI) | Ref. | 0.04 (-0.17, 0.25) | -0.04 (-0.25, 0.17) | 0.686 |
| **Stilbenes** | | | | |
| Model 1. β (95%CI) | Ref. | -0.21 (-0.44, 0.02) | -0.08 (-0.30, 0.14) | 0.985 |
| Model 2. β (95%CI) | Ref. | -0.29 (-0.52, -0.05) | -0.41 (-0.69, -0.14) | 0.019 |
| Model 3. β (95%CI) | Ref. | -0 26 (-0.50, -0.03) | -0.38 (-0.66, -0.11) | 0.027 |
| Model 4. β (95%CI) | Ref. | -0.28 (-0.51, -0.04) | -0.42 (-0.71, -0.14) | 0.018 |
| **Lignans** | | | | |
| Model 1. β (95%CI) | Ref. | 0.01 (-0.21, 0.22) | -0.04 (-0.25, 0.18) | 0.723 |
| Model 2. β (95%CI) | Ref. | 0.02 (-0.20, 0.23) | -0.02 (-0.23, 0.19) | 0.843 |
| Model 3. β (95%CI) | Ref. | 0.02 (-0.19, 0.23) | -0.03 (-0.25, 0.18) | 0.747 |
| **Tyrosols** | | | | |
| Model 1. β (95%CI) | Ref. | -0.09 (-0.31, 0.13) | -0.11 (-0.33, 0.10) | 0.350 |
| Model 2. β (95%CI) | Ref. | -0.11 (-0.33, 0.11) | -0.15 (-0.38, 0.07) | 0.209 |

| | | | | |
|---|---|---|---|---|
| Model 3. β (95%CI) | Ref. | -0.11 (-0.32, 0.11) | -0.14 (-0.36, 0.09) | 0.275 |
| **Alkylphenols** | | | | |
| Model 1. β (95%CI) | Ref. | -0.02 (-0.25, 0.20) | 0.07 (-0.15, 0.29) | 0.403 |
| Model 2. β (95%CI) | Ref. | 0.03 (-0.19, 0.25) | 0.23 (0.01, 0.46) | 0.031 |
| Model 3. β (95%CI) | Ref. | 0.03 (-0.19, 0.26) | 0.24 (0.01, 0.47) | 0.026 |

* The unit have been added to the original values to later be logarithmically transformed (transformation log (x + 1))
**CACS:** Coronary Calcium Agatston Score; **CI**: Confidence Interval; **OR**: Odds Ratio.
**Model 1**: Logistic regression model adjusted for age and total energy intake.
**Model 2**: As in Model 1 and additionally adjusted for marital status, education, smoking, physical activity, sleep duration during weekdays and during the weekend, alcohol consumption, total fiber intake, body mass index, and diabetes.
**Model 3**: As in Model 2 and additionally adjusted for LDL and HDL cholesterol and systolic and diastolic blood pressure.
**Model 4**: As in Model 3 and flavonoids and stilbenes were mutually adjusted.
