# Supplementary material for: The intake of flavonoids, stilbenes, and tyrosols, mainly consumed through red wine and virgin olive oil, is associated with lower carotid and femoral subclinical atherosclerosis and coronary calcium
Source: Eur J Nutr. 2022 Mar 7;61(5):2697–709. doi: 10.1007/s00394-022-02823-0 (PMC9279214; doi:10.1007/s00394-022-02823-0)

Supplemental Figure 1. Flow chart of the final sample of the subjects recruited in the imaging AWHS Study.

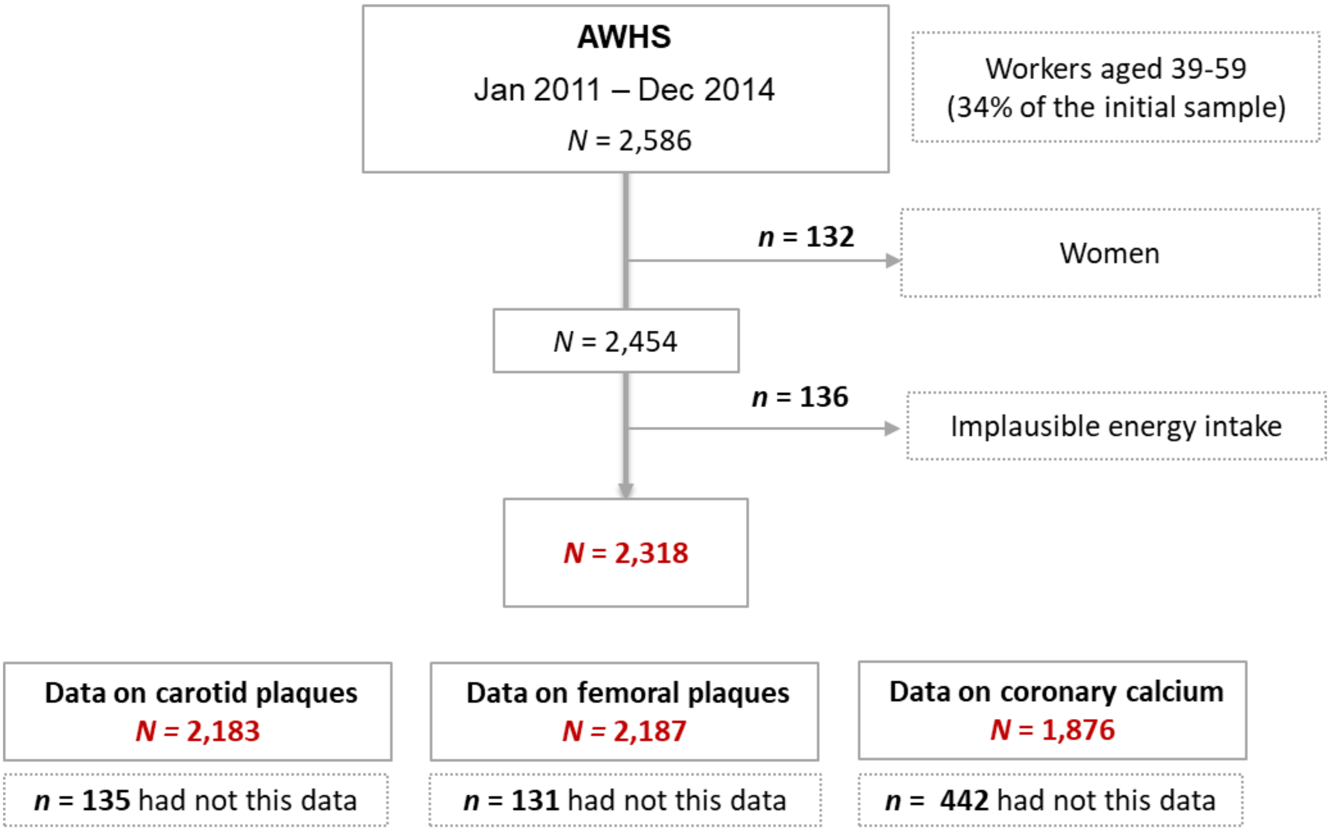

Supplement: Supplementary file 2 — Supplementary file2 (PDF 36 KB) [file 394_2022_2823_MOESM2_ESM.pdf]
